# The Relationship Between the Distribution of Training Intensity and Performance of Kayak and Canoe Sprinters: A Retrospective Observational Analysis of One Season of Competition

**DOI:** 10.3389/fspor.2021.788108

**Published:** 2022-01-05

**Authors:** Manuel Matzka, Robert Leppich, Hans-Christer Holmberg, Billy Sperlich, Christoph Zinner

**Affiliations:** ^1^Integrative and Experimental Exercise Science and Training, University of Würzburg, Würzburg, Germany; ^2^Software Engineering Group, Department of Computer Science, University of Würzburg, Würzburg, Germany; ^3^Department of Health Sciences, Luleå University of Technology, Luleå, Sweden; ^4^Department of Sport, University of Applied Sciences for Police and Administration of Hesse, Wiesbaden, Germany

**Keywords:** kayaking, training intensity distribution, training zones, high-volume training, pyramidal intensity distribution, water sport

## Abstract

**Purpose:** To evaluate retrospectively the training intensity distribution (TID) among highly trained canoe sprinters during a single season and to relate TID to changes in performance.

**Methods:** The heart rates during on-water training by 11 German sprint kayakers (7 women, 4 men) and one male canoeist were monitored during preparation periods (PP) 1 and 2, as well as during the period of competition (CP) (total monitoring period: 37 weeks). The zones of training intensity (Z) were defined as Z1 [<80% of peak oxygen consumption (VO_2peak_)], Z2 (81–87% VO_2peak_) and Z3 (>87% VO_2peak_), as determined by 4 × 1,500-m incremental testing on-water. Prior to and after each period, the time required to complete the last 1,500-m stage (all-out) of the incremental test (1,500-m time-trial), velocities associated with 2 and 4 mmol·L^−1^ blood lactate (v2_[BLa]_, v4_[BLa]_) and VO_2peak_ were determined.

**Results:** During each period, the mean TID for the entire group was pyramidal (PP1: 84/12/4%, PP2: 80/12/8% and CP: 91/5/4% for Z1, Z2, Z3) and total training time on-water increased from 5.0 ± 0.9 h (PP1) to 6.1 ± 0.9 h (PP2) and 6.5 ± 1.0 h (CP). The individual ranges for Z1, Z2 and Z3 were 61–96, 2–26 and 0–19%. During PP2 VO_2peak_ (25.5 ± 11.4%) markedly increased compared to PP1 and CP and during PP1 v2_[bla]_ (3.6 ± 3.4%) showed greater improvement compared to PP2, but not to CP. All variables related to performance improved as the season progressed, but no other effects were observed. With respect to time-trial performance, the time spent in Z1 (*r* = 0.66, *p* = 0.01) and total time in all three zones (*r* = 0.66, *p* = 0.01) showed positive correlations, while the time spent in Z2 (*r* = −0.57, *p* = 0.04) was negatively correlated.

**Conclusions:** This seasonal analysis of the effects of training revealed extensive inter-individual variability. Overall, TID was pyramidal during the entire period of observation, with a tendency toward improvement in VO_2peak_, v2_[bla]_, v4_[bla]_ and time-trial performance. During PP2, when the COVID-19 lockdown was in place, the proportion of time spent in Z3 doubled, while that spent in Z1 was lowered; the total time spent training on water increased; these changes may have accentuated the improvement in performance during this period. A further increase in total on-water training time during CP was made possible by reductions in the proportions of time spent in Z2 and Z3, so that more fractions of time was spent in Z1.

## Introduction

For decades, both coaches and researchers have sought to improve the performance of endurance athletes by determining the optimal schedule for training intensity (Stöggl and Sperlich, 2015). This intensity is routinely categorized into 3–5 different zones, depending on the sport, the approach of the sports federation concerned to diagnosing performance, monitoring of training, and the availability of appropriate technology (Stöggl and Sperlich, 2015).

This partitioning into zones allows the fractional distribution of exercise intensity (e.g., within a training session or a meso- and macrocycle) to be quantified. Previously, the following three-zone model has been employed most widely: (i) Zone (Z) 1, in which the intensity is at or below the aerobic threshold; (ii) Z2, with an intensity between the aerobic and anaerobic thresholds; and (iii) Z3, involving an intensity above the anaerobic threshold (Esteve-Lanao et al., 2005; Seiler and Kjerland, 2006; García-Pallarés et al., 2009, 2010; Plews et al., 2014; Baldassarre et al., 2019; Bellinger et al., 2020). [For further details concerning the concepts of aerobic and anaerobic thresholds, please see Faude et al. (2009)]. With this model, exercise performed predominantly in Z1 is often referred to as low-intensity continuous exercise or aerobic endurance training, Z2 as “threshold training” and Z3 as high-intensity interval training (Stöggl and Sperlich, 2015).

The training intensity distribution (TID) may vary between sports and seasons (i.e., the periods of preparation, transition, tapering, and competition) (Treff et al., 2017; Kenneally et al., 2020). A combination of all three zones involving substantial training in Z1 with relatively decreasing proportions of Z2 and Z3 is referred to as pyramidal (Stöggl and Sperlich, 2015); whereas a TID where the relationship between training in these zones is Z1 > Z3 >Z2, with a polarization index of >2.0 arbitrary units (Treff et al., 2019), is defined as “polarized” (Seiler and Kjerland, 2006).

Most retrospective analyses of the TID of athletes engaged in various endurance sports, such as rowing (Hartmann et al., 1990; Guellich et al., 2009; Plews et al., 2014), cycling (Lucía et al., 2000; Schumacher and Mueller, 2002; Zapico et al., 2007; Neal et al., 2011), swimming (Mujika et al., 1995; Baldassarre et al., 2019), running (Esteve-Lanao et al., 2005), triathlon (Neal et al., 2013) and cross-country skiing (Torvik et al., 2021), have revealed a pyramidal structure, with > 70% of the training being performed in Z1. However, some retrospective analyses do report utilization of a polarized TID by successful cross-country skiers (Seiler and Kjerland, 2006; Sandbakk et al., 2011; Tønnessen et al., 2014; Schmitt et al., 2020), runners (Billat et al., 2001) and rowers (Bourgois et al., 2014).

Interestingly, almost all investigations of TID to date have involved individual sports where the leg muscles produce most of the propulsion (e.g., cycling and running) (Lucía et al., 2000; Schumacher and Mueller, 2002; Billat et al., 2003; Zapico et al., 2007). There are relatively few reports of this nature about sports where both the arms and legs are directly involved in producing propulsion (e.g., cross-country skiing, rowing, swimming, and triathlon) (Hartmann et al., 1990; Mujika et al., 1995; Neal et al., 2011; Plews et al., 2014; Baldassarre et al., 2019; Torvik et al., 2021).

Sprint kayakers propel both the mass of their body and of the boat against the resistance of the water, which places extensive demands on the endurance of the relatively small upper-body muscles (Ualí et al., 2012). Upper- and lower-body muscles differ substantially with respect to mass, fiber composition, extraction of oxygen (Calbet et al., 2005), and the contractile properties of muscle fibers (Gejl et al., 2021), as well as in the oxidation of glucose and lipid (van Hall et al., 2003; Calbet et al., 2005; Helge, 2010; Zinner et al., 2016; Ørtenblad et al., 2018). This indicates that the training required to achieve optimal adaptations in the upper and lower bodies differs both qualitatively and quantitatively. Furthermore, sport-specific differences in the demands of competition, the constraints of movement (e.g., weight-bearing vs. seated, concentric vs. plyometric work by the muscle-tendon complex), and the individual tolerance for training exert a considerable impact on training volume (Esteve-Lanao et al., 2017), as well as the amount of high-intensity work that can be tolerated (Sandbakk et al., 2021). These considerations indicate that the optimal long-term TID for kayak and canoe sprinting should differ from that for other sports involving primarily the legs.

To date, only two prospective reports have evaluated the utilization of different TID by elite sprint kayakers (García-Pallarés et al., 2009, 2010), revealing that for these athletes an emphasis on Z2 or Z3 is effective and comparable to the pyramidal or polarized TID in other sports in which the legs play a predominant role. However, prospective training interventions such as these are relatively short and often alter the typical training schedule in an artificial manner. To the best of our knowledge, the TID of highly trained sprint kayakers and canoeists during the periods of preparation, transition and competition and its association with changes in performance have yet to be analyzed retrospectively. Accordingly, the present study was designed to assess these aspects of the training of such elite athletes.

## Materials and Methods

### Experimental Design

**Figure 1:**
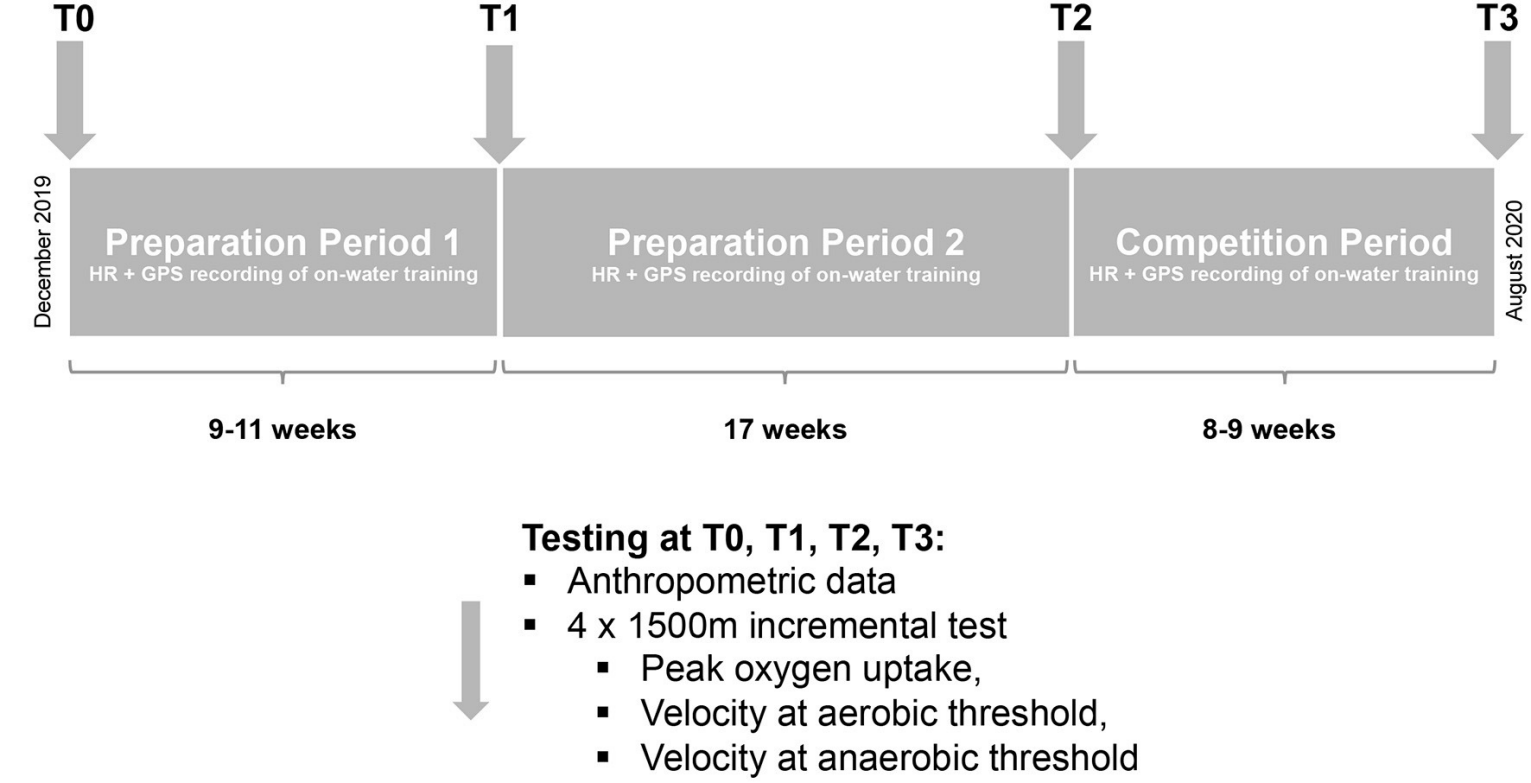
The overall study design. HR, heart rate; GPS, global positioning system; T, Time-point of testing.

This retrospective observational study was conducted from December 2019 until August 2020, i.e., one season for competitive kayakers and canoeists. Figure 1 illustrates the time course, methods involved, and parameters obtained.

This season consisted of two preparation periods (PP1, 9–11 weeks and PP2, 17 weeks), together with the period of competition (CP, 7–8 weeks). Before and after each of these periods, the performance of all subjects was tested. The study period ended for each athlete with the German national championships, which took place during the first or second week of August, depending on age. PP2 overlapped in part with the period during which the first measures designed to counter the COVID-19 pandemic were enacted in Germany.

### Participants

Initially, 21 athletes were enrolled in this study. Of these, 4 male and 7 female flatwater sprint kayakers, as well as one male canoeist (who was also included because the training regimes and diagnostic procedures for kayaking and canoeing are similar) provided complete information for at least one of the three periods, whereas the remaining 9 could not, due to, e.g., illness, injury, COVID-19 related quarantine, etc. and were therefore excluded. 4 women and 3 men, all kayakers, provided complete information for the entire period of investigation. The numbers of participants who provided complete HR data for each period and who were thereby included in the analysis were as follows: for PP1: 10 kayakers (7 women, 3 men) and the male canoeist (subjects 1, 2, 3, 4, 6, 7, 8, 9, 10, 11, 12); PP2: 8 kayakers (5 women, 3 men) (subjects 2, 3, 4, 6, 7, 8, 9, 11); and CP: 9 kayakers (5 women, 4 men) (subjects 2, 3, 4, 5, 6, 7, 9, 11, 12).

These athletes were recruited from three different training facilities in Germany, all of which are official performance centers of the German and/or North Rhine-Westphalian Canoe Federation. Their key anthropometric, physiological and performance characteristics are documented in Table 1.

**Table 1:** Characteristics of the participants before (Pre) and at the end of (Post) the period of observation.

**Parti cipant Nr**.	**Discipline**	**Sex**	**Age (years)**		**Height (cm)**		**Body mass (kg)**		**Body mass index [kg·(m^2^)^−1^]**		**Peak oxygen uptake (ml·min^−1^)**		**Personal best times at** **national championships 2020 [s]**		
			**Pre**	**Post**	**Pre**	**Post**	**Pre**	**Post**	**Pre**	**Post**	**Pre**	**Post**	**200 m**	**500 m**	**1,000 m**
1	C	m	17	18	178	178	74.8	74.9	23.6	23.6	3,391	4,047	<45	<120	<272
2	K	m	17	18	182	182	80.0	80.9	24.2	24.4	3,635	4,217	<39	<106	<225
3	K	m	17	18	195	198	85.9	88.9	22.6	22.7	4,452	5,427	<41	<106	<226
4	K	m	22	23	179	179	72.8	75.3	22.7	23.5	4,466	4,727	n.d.	<106	<223
5	K	m	15	16	180	187	71.7	76.7	22.1	21.9	3,122	4,069	n.d.	<108	<231
6	K	w	16	17	170	170	67.6	66.3	23.4	22.9	2,576	2,975	<43	<120	<262
7	K	w	17	18	174	174	71.2	76.5	23.5	25.3	2,700	3,478	<48	<124	<265
8	K	w	20	21	171	171	73.3	73.2	25.1	25.0	3,381	3,684	<45	<122	<262
9	K	w	15	15	167	167	60.5	62.7	21.7	22.5	2,729	2,881	n.d.	<125	<258
10	K	w	15	16	175	175	61.6	64.5	20.1	21.1	2,836	3,197	<46	n.d.	<272
11	K	w	16	16	177	177	69.7	75.4	22.3	24.1	2,555^*^	3,865	<45	<122	<258
12	K	w	16	17	166	166	66.4	68.0	24.1	24.7	2,985	3,038^#^	<47	<125	<266
Mean			16.9	17.8	176	177	71.3	73.6	23.0	23.5	3,236	3,800	43.6	116.1	251.1
SD			2.1	2.3	7.8	9.0	7.1	7.4	1.2	1.3	666	763	3.1	8.3	19.5

* *This value is based on the test performed after preparatory period 1, since the data from initial testing were unreliable. This value is provided simply to enable evaluation of the individual's development and was not included in the analysis of TID*.

# *This value is based on the test performed before the period of competition, since the data from the final testing were unreliable. This value is provided simply to enable evaluation of the individual's development and was not included in the analysis of TID*.

*C, Canoe; K, Kayak; n.d., not determined; m, man; w, woman*.

During the season examined, 5 of the 12 participants included in the statistical analyses were members of the German Development Team, one had belonged to the German National Under 23 Team during the preceding year and 6 were members of the Western German Regional Team. All had competed in kayaking or canoeing for at least 6 years and during the study period were competing at the highest national level.

All of these athletes had undergone testing frequently throughout their career and were therefore quite familiar with all testing procedures employed. After being informed in detail about these procedures, as well as the risks and benefits of this investigation, all of the athletes and their legal guardians consented in writing to their participation. All procedures were approved by the institute's ethics committee and conducted in accordance with the Declaration of Helsinki.

### Monitoring of Training

#### Heart Rate, Distance, and Duration

The heart rate (HR) of each athlete during each training session, as well as the distance and duration, were monitored with a watch receiving Global Positioning System (GPS) data (M430, Polar Electro OY, Kempele, Finland) and stored online (Polar Flow, Polar Electro OY). The athletes initiated recording at the beginning of warm-up and stopped immediately after cool-down. All sets of data found by visual inspection to contain artifacts (e.g., flatline) or be incomplete due to technical problems (e.g., low battery, inaccurate monitoring of HR or GPS data, etc.) were excluded from further analysis. In addition, an experienced coach compared the number of sessions recorded with the online training diary of each athlete to detect potential discrepancies. Table 2 shows the mileage indicated by the HR monitor and included in the analysis as a percentage of the mileage reported by each athlete in his/her online training diary. The lower limit for inclusion was set at 70%. Furthermore, the raw data provided by the HR monitor (Polar Flow software) were inspected by experienced coaches for flawed or unreasonable values, which were also removed. Following these quality controls, the total amount of time spent in each zone during each training session was calculated.

**Table 2:** The mileage indicated by the GPS watch and included in data analysis as a percentage of the mileage reported in the online diary by each individual athlete for each training period.

**Participant Nr**.	**Training period**		
	**PP1**	**PP2**	**CP**
1	100%		
2	100%	100%	71%
3	100%	100%	100%
4	88%	100%	94%
5	90%	78%	100%
6	100%	100%	100%
7	86%	89%	
8	78%	95%	100%
9	97%		
10	100%	100%	100%
11	94%		100%
12			100%

#### Definition of the Different Intensity Zones

Utilizing the categorization proposed by Seiler (2010), Zone 1 (Z1), 2 (Z2) and 3 (Z3) were defined as when the HR corresponded to values observed at an exercise intensity of 60–80%, 81–87% and ≥87% of peak oxygen consumption (VO_2peak_), respectively. VO_2peak_ was obtained from the incremental step-test performed prior to each period.

#### Polarization Index

To quantify the individual levels of periodization, we calculated a Polarization-Index (Pol-Index in arbitrary units) based on the time spent in each intensity zone, as described in detail previously (Treff et al., 2017, 2019). The Pol-Index was calculated as:

*Pol-Index (a.u.)* = *log (Z1*·*Z2*^−1^ ·*Z3)*.

#### Incremental Testing

All testing on-water was conducted on the regatta course in Duisburg-Wedau (Germany), the venue for several international canoe sprint championships, as well as for the World Cup series arranged by the International Canoe Federation (ICF). The participants were requested to refrain from all physical exercise for 12 hours and from exhausting exercise for 48 hours prior to the experimental sessions, as well as to maintain their normal diet. In addition, the athletes were instructed to avoid food intake for two hours prior to testing, to arrive well-hydrated state and to refrain from consuming caffeine on the day of testing. Their physical activity, diet and hydration were assessed with questionnaires administered prior to the tests.

The incremental test protocol consisted of 1,500-m trials on water at 70, 80, and 90% of peak HR (HR_peak_), as well as an all-out effort, as described in detail previously (Matzka et al., 2021). Each test involved a 180° turn at the half-way mark (i.e., after 750 m), in attempt to minimize the influence of wind and waves on performance. The wind speed, air temperature, water temperature, humidity, and atmospheric pressure on the days of testing are shown in Table 3. The water temperature was measured with a thermometer (TFA Dostmann Marbella, TFA Dostmann GmbH and Co. KG, Germany) and all other data concerning the weather collected by the weather station located at the regatta course (Duisburg-Wedau, Germany). The time-point for each change in direction was determined on the basis of the GPS data.

**Table 3:** Weather conditions during each day of testing.

	**T0**	**T1**	**T2**	**T3**
Air temperature (°C)	4.0 ± 2.2	6.5 ± 0.7	24.0 ± 1.8	25.5 ± 2.3
Wind speed (km·h^−1^)	9.1 ± 2.2	25.7 ± 4.3	17.6 ± 4.2	17.4 ± 7.6
Water temperature (°C)^*^	7.5	6.3	18.4	20.7
Humidity (%)	88 ± 7	70 ± 6	51 ± 7	64 ± 9
Atmospheric pressure (hPa)	1,024 ± 0.3	1,019 ± 1.1	1,006 ± 0.8	1,009 ± 0.5

*T0, immediately prior to PP1; T1, immediately prior to PP2; T2, immediately prior to CP; T3, immediately after CP*.

* *In our experience, water temperature does not change during the duration of our testing to an extent that would exert a measurable impact on performance. Therefore, this temperature was measured only once, half-way through the period of testing on each day*.

As members of the Canoe Federation of North-Rhine-Westphalia, all of our subjects were highly experienced in performing this particular protocol, which has been employed by this federation for decades and the results of which correlate closely with kayaking performance during ergometric testing in the laboratory (Matzka et al., 2021). Indeed, this extensive experience was considered important for achieving optimal reliability and validity. In fact, in connection with previous investigations employing a protocol that differed only slightly from our own with respect to the number, duration, and intensity of incremental steps, the reliability and validity of measurements of HR, oxygen consumption (VO_2_), blood lactate and stroke rate were all reported to be acceptable to excellent (Carrasco Páez et al., 2010; Winchcombe et al., 2019; Matzka et al., 2021).

The HR utilized for the first test was the HR_peak_ obtained 6–8 weeks before by the Western German Canoe Federation utilizing the same incremental on-water test protocol and each subsequent test utilized the maximal HR (considered to be HR_peak_) attained during the preceding test. The 30–45 s interval between successive steps was required to sample capillary blood from the earlobe. All participants received continuous visual feedback from the HR monitor (Polar Wear Link System and V800 HR Monitor, Polar Electro OY, Kempele, Finland) mounted directly in front of them, which averaged this value every second.

During each stage stroke rate was self-selected. Maximal exhaustion was considered to have been reached when three of the four following criteria were met: (1) a plateau in oxygen uptake (i.e., an increase of ≤ 1.0 ml·kg^−1^·min^−1^, despite an increase in velocity); (2) a respiratory exchange ratio >1.1; (3) a HR within ± 5% of the HR_peak_ obtained from previous incremental test; and (4) a peak blood lactate concentration ≥ 6 mmol·L^−1^. In addition, the rating of perceived exertion (RPE) on the 6–20-point Borg scale (Borg, 1970) had to be ≥ 18.

For analysis of lactate (Lactate Pro 2, Arkray KDK, Kyoto, Japan), capillary blood was sampled from the right earlobe at baseline and following each step. The velocities associated with blood lactate concentrations of 2 (v2_[BLa]_) and 4 mmol·L^−1^ (v4_[BLa]_) were determined by linear interpolation between the two nearest points, as previously described (Zinner et al., 2018). At these same time-points, RPE was assessed employing the 6–20-point Borg scale (Borg, 1970).

Oxygen uptake was monitored continuously by an open-circuit breath-by-breath analyzer (MetaMax 3B, Cortex Biophysik, Leipzig, Germany), employing standard algorithms to compensate for the time delay between gas consumption and the signal. This analyzer was calibrated prior to each test with both 15.8 and 5% O_2_ in N_2_ (Praxair, Düsseldorf, Germany), i.e., concentrations that cover the range of the expected fractional concentration of O_2_. The volume sensor was calibrated with a precision 3-L syringe (Cortex Biophysik, Leipzig, Germany). Average respiratory values were calculated for the last 120 s of each individual increment. The highest VO_2_ (averaged over 30-s intervals) was considered to be VO_2peak_.

### Statistical Analyses

Application of the Shapiro-Wilk test revealed that all data were distributed normally, making transformation unnecessary. For analysis of differences between the amount of time spent in each of the three training zones during each period of training, the use of repeated-measures ANOVA would have required exclusion of incomplete data sets, which would have reduced statistical power. This is also the case with respect to analysis of the interactions between training period and the dependent variables VO_2peak_, v2_[Bla]_, v4_[Bla]_ and performance time in connection with the all-out 1,500-m time-trial. Consequently, we chose to utilize a linear-mixed model for these analyses.

As shown in Table 4, data for eleven, ten, nine and eight athletes could be collected at T0, T1, T2, T3, respectively and were included in the analyses for PP1, PP2 and CP. For comparison of the amount of time spent in the different training zones during each training period, these periods were the fixed factor and the subjects the random factor. For analysis of the effects of the training period on the different parameters of related to performance, the four time-points of incremental testing were the fixed factor and the subjects the random factor. When a significant fixed-effect was found, pair-wise comparison of each training period and time-point of testing was also performed. Family-wise error was corrected for by utilizing the Bonferroni *post-hoc* test. The Statistical Package for Social Science (version 26; IBM Corp., Armonk, NY) was utilized for these analyses.

**Table 4:** Physiological characteristics and parameters related to performance at the four time-points for testing.

**Variable**	**T0** **(***n*** = 11)**	**T2** **(***n*** = 10)**	**T3** **(***n*** = 9)**	**T4** **(***n*** = 8)**
1,500-m time-trial performance (s)	419 ± 27 (*n* = 11)^*^	417 ± 30 (*n* = 10)^*^	400 ± 33 (*n* = 9)^*^	394 ± 33 (*n* = 8)^*^
VO_2peak_ (ml·min^−1^)	3,315 ± 694 (*n* = 10)^*^	3,212 ± 738 (*n* = 10)^*^	4,001 ± 1,136 (*n* = 8)^*^	3,954 ± 861 (*n* = 8)^*^
v2_[Bla]_ (km·h^−1^)	11.67 ± 0.76 (*n* = 11)^*^	12.14 ± 0.78 (*n* = 10)^*^	12.05 ± 0.78 (*n* = 9)^*^	12.48 ± 0.74 (*n* = 8)^*^
v4_[Bla]_ (km·h^−1^)	12.33 ± 0.79 (*n* = 11)^*^	12.69 ± 0.86 (*n* = 10)^*^	13.02 ± 0.82 (*n* = 9)^*^	13.34 ± 0.82 (*n* = 8)^*^

*T0, immediately prior to PP1; T1, immediately prior to PP2; T2, immediately prior to CP; T3, immediately after CP*.

*1,500-m time-trial performance, performance in connection with the all-out 1,500-m trial during the incremental step test; VO_2peak_, peak oxygen uptake; v2_[Bla]_, velocity associated with a blood lactate concentration of 2 mmol·L^−1^; v4_[Bla]_, velocity associated with a blood lactate concentration of 4 mmol·L^−1^*.

* *Only those athletes for whom HR monitoring for the period indicated was complete and data from the incremental testing at the four different time-points reliable were included in each analysis. This explains why the numbers of participants analyzed at each time-point and, to a certain extent, with respect to the different variables, are not the same*.

For analysis of potential correlations between the various physiological characteristics and parameters related to performance, on the one hand, and the time spent in each individual zone, the total time spent in all three zones, and the Polarization Index, on the other hand, repeated-measures correlation for determination of within-individual association regarding paired measures assessed on several occasions for a number of different individuals was applied using the R software, as described previously (Bakdash and Marusich, 2017). As in the case with the linear mixed model, this analysis included only the pre- and post-values of the physiological measures of performance for each individual training period and athlete, if the data were complete (Table 4). The descriptors for the correlation values obtained were as follows: *r* < 0.1—very small, 0.1 ≤ *r* < 0.3—small, 0.3 ≤ *r* < 0.5—moderate, 0.5 ≤ *r* < 0.7—large, 0.7 ≤ *r* < 0.9—very large, *r* ≥ 0.9—nearly perfect (Hopkins, 2002).

For all statistical analyses significance was defined as *p* ≤ 0.05. All values concerning the entire group are presented as means with standard deviations.

## Results

**Figure 2:**
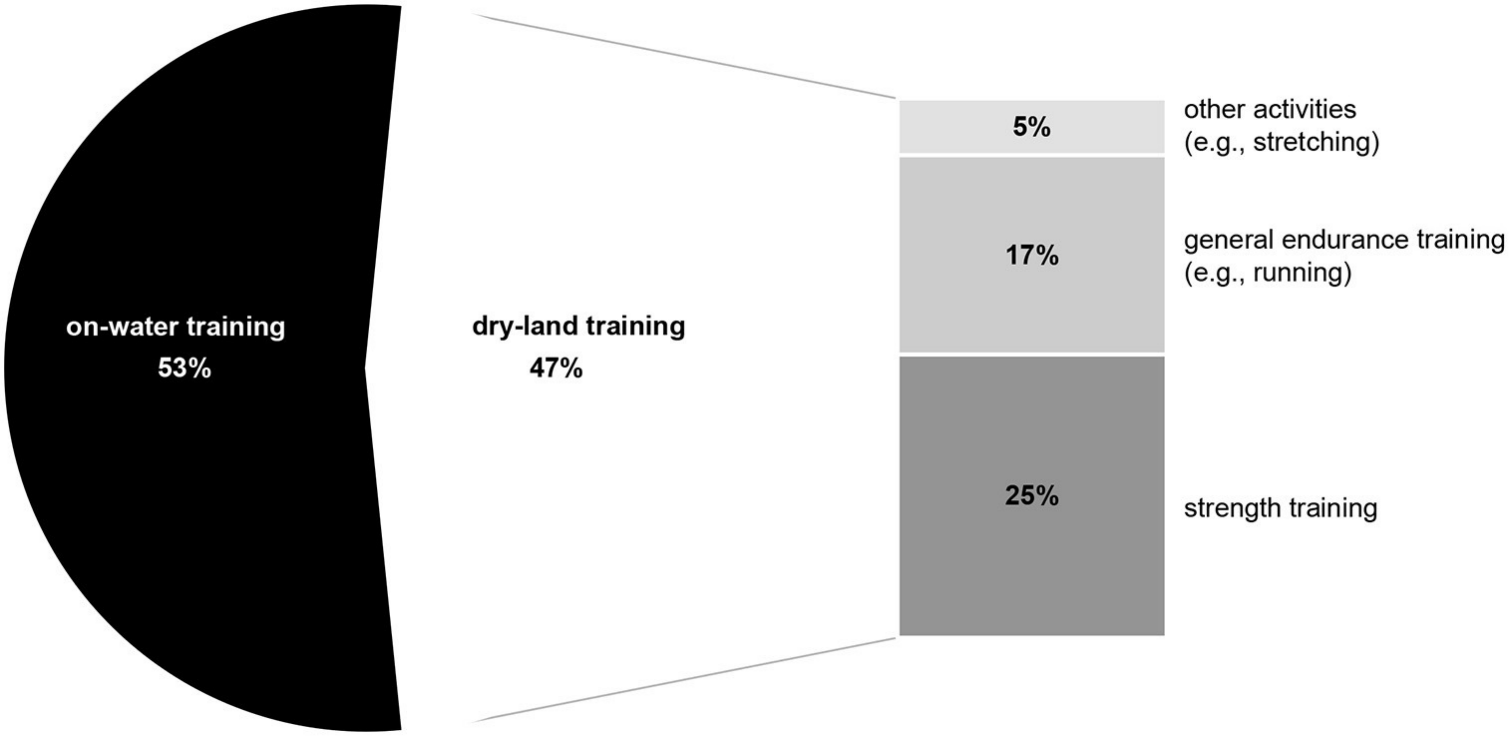
Overall distribution of the different aspects of training during the period of observation.

The current analysis included 2,132 individual sessions of training during a period of 34–37 weeks, covering ~17,900 km and lasting for a total of about 2,000 h. As indicated by the athletes' online training diaries, the average overall time spent training each week was 12.3 ± 1.8 h, including 6.5 ± 1.2 h on-water, 3.0 ± 0.5 h strength training, 2.1 ± 0.5 h general endurance training (e.g., running, swimming, cycling) and 0.6 ± 0.4 h of other activities (e.g., stretching, etc.) (Figure 2). The number of weekly training sessions on water increased from 5.6 ± 0.8 during PP1 to 6.9 ± 0.8 during PP2 and 8.7 ± 1.5 h during CP, with the corresponding total training time on-water increasing from 5.0 ± 0.9 h to 6.1 ± 0.9 h and, finally, 6.5 ± 1.0 h.

**Figure 3:**
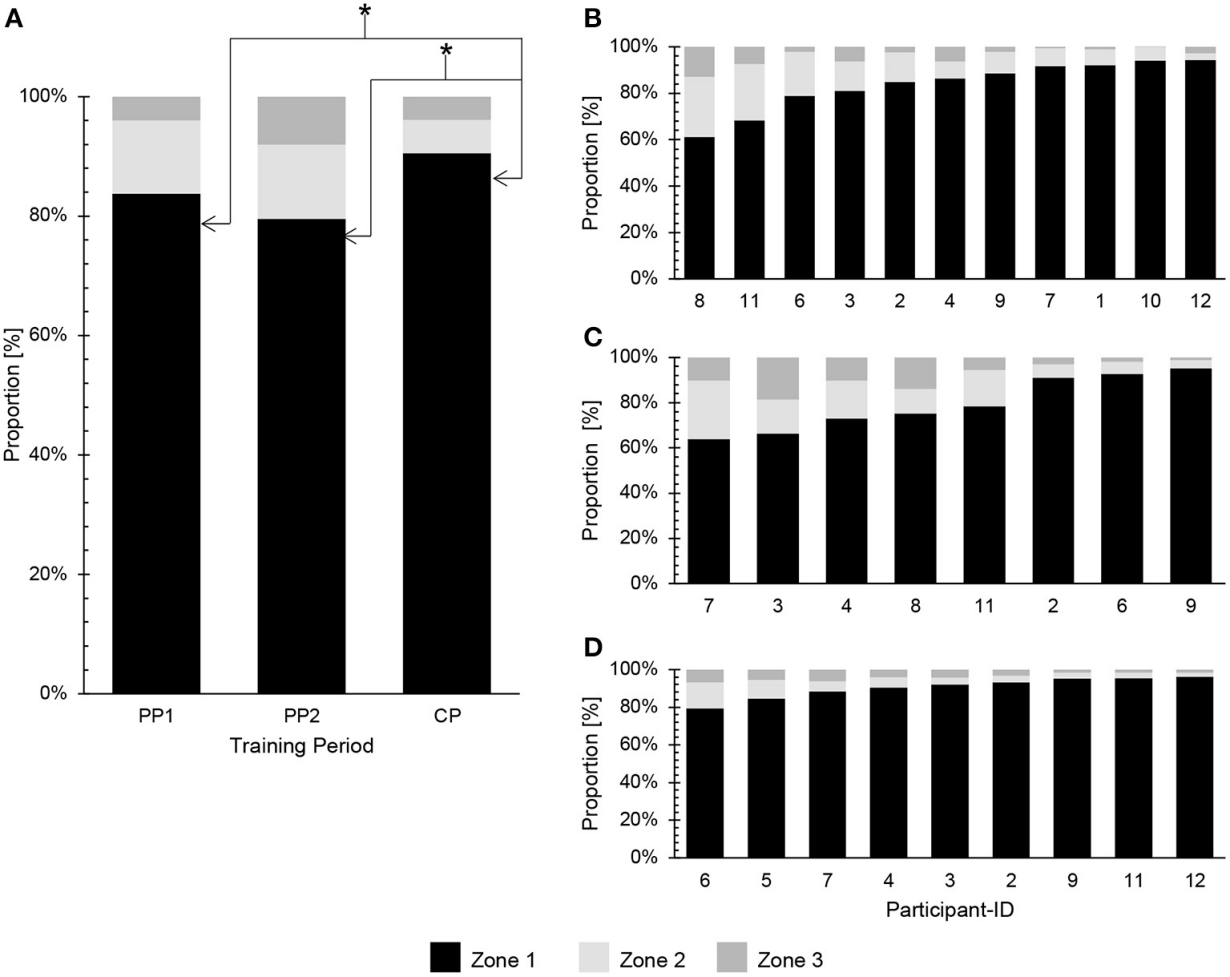
Percentage distribution of training intensity during the three periods. **(A)** Group mean during PP1, PP2 and CP. **(B)** Individual values during PP1. **(C)** Individual values during PP2. **(D)** Individual values during CP. *Significant difference with respect to the percentage of time spent in Zone 1 between PP1 and CP (*p* < 0.01), as well as between PP2 and CP (*p* = 0.03).

**Figure 4:**
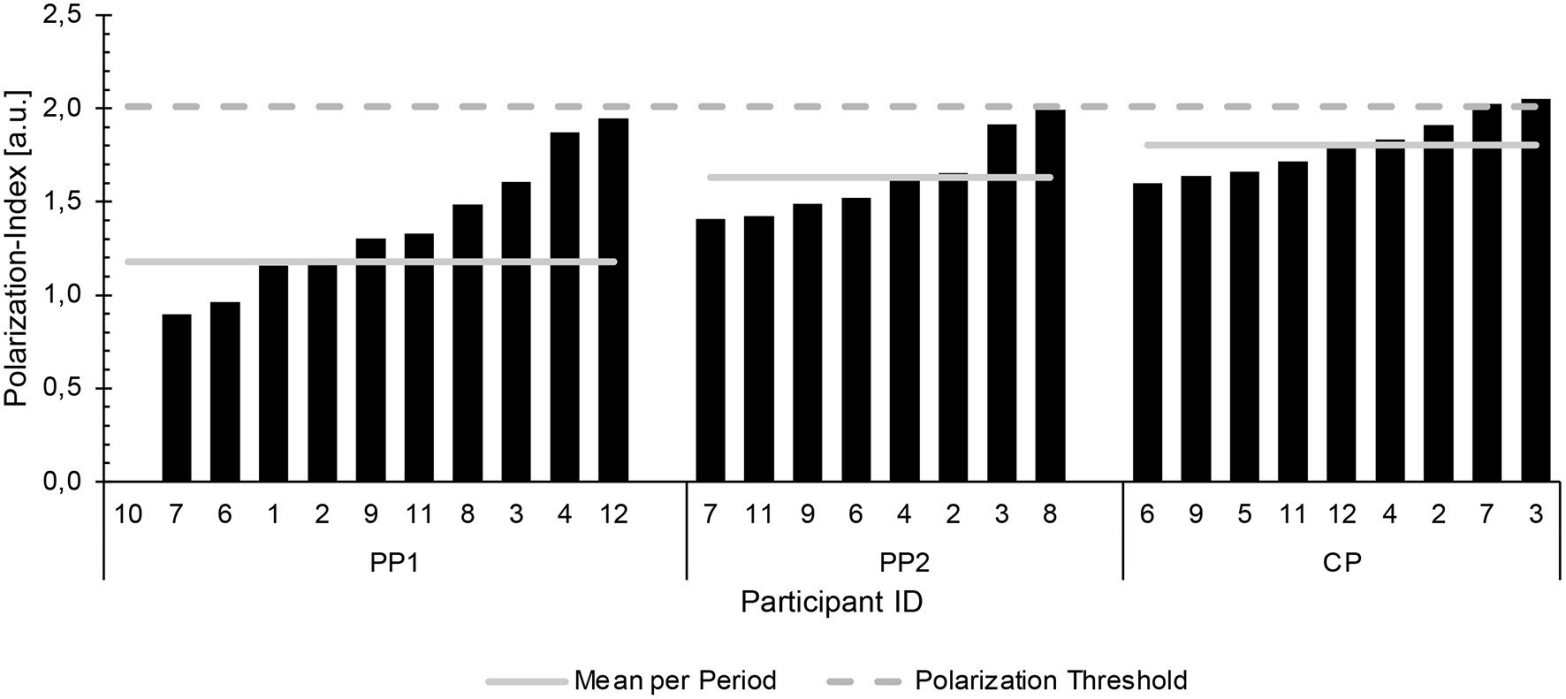
The Polarization Indices (in arbitrary units) for each period and athlete analyzed. PP, preparatory period; CP, competition period.

The TIDs for each period and each participant are summarized in Figure 3, with Figure 4 documenting the corresponding Pol-Indices.

For the entire season the fractions of training performed in Z1, Z2 and Z3 were 85 ± 11, 10 ± 7 and 5 ± 5%, respectively (Pol-Index: 1.6 ± 0.3). For PP1, the corresponding values were 84 ± 11, 12 ± 8 and 4 ± 4% (Pol-Index: 1.4 ± 0.4); in the case of PP2, 80 ± 12, 12 ± 7 and 8 ± 6% (Pol-Index: 1.4 ± 0.2); and during CP 91 ± 5, 5 ± 4 and 4 ± 2% (Pol-Index: 1.8 ± 0.2) (Figures 3, 4). Application of linear mixed-model analysis revealed an effect of the training period on Z1 (*p* < 0.01), but not Z2 (*p* = 0.15) or Z3 (*p* = 0.08). Bonferroni-adjusted *post-hoc* analysis of Z1 showed differences between PP1 (211 ± 47 min per week) and CP (310 ± 57 min per week) [*p* < 0.01, *d* = −1.91, 95%CI (−2.88 to −0.78)], as well as between PP2 (237 ± 54 min per week) and CP [*p* = 0.03; *d* = −1.30, 95%CI (−2.27 to −0.20)].

The corresponding ranges were as follows: During PP1 the proportions of Z1, Z2 and Z3 were 61–94, 3–26 and 0–13%, respectively (Pol-Index: 0.9–1.9); for PP2 64–95, 4–26 and 1–19% (Pol-Index: 1.4–2.0); and in the case of CP 81–96, 2–13 and 1–6% (Pol-Index: 1.6–2.0). During PP1 11 of the athletes exhibited a pyramidal TID (Pol-Index <2.0). The corresponding value for PP2 was 6, with two (participants 3 and 8) exhibiting a more polarized TID (Pol-Index: 1.91–1.99), with higher proportions of Z3 than Z2. During the CP, participant 6 displayed a polarized TID (Pol-Index: 2.04), while the TID of the other 8 was pyramidal (Pol-Index: 1.58–1.94).

Table 4 summarizes the values for physiological characteristics and parameters related to performance prior to and after each training period.

Application of a linear mixed model revealed that the period of training exerted an impact on changes in v2_[bla]_ (*p* = 0.03) and VO_2peak_ (*p* > 0.01). Bonferroni-adjusted *post-hoc* analysis showed differences between changes in v2_[bla]_ during PP1 (3.62 ± 3.43%) and PP2 (−0.84 ± 2.16%) [*p* = 0.04, *d* = 1.48, 95%CI (0.33 – 2.47)], but not between PP2 and CP (2.67 ± 2.37%) [*p* = 0.08, *d* = −1.53, 95%CI (−2.57 to −0.30)] or PP1 and CP [*p* = 1.00, *d* = 0.31, 95%CI (−0.64 to 1.23)]. Regarding VO_2peak_, such analysis revealed differences in the changes during PP1 (−3.11 ± 5.94%) and PP2 (25.48 ± 11.38%) [*p* < 0.01, *d* = −3.06, 95%CI (−4.34 to −1.42)], as well as between PP2 and CP (0.34 ± 12.06%) [*p* = 0.04, *d* = 1.90, 95%CI (0.52–3.01)], but not between PP1 and CP [*p* = 1.00, *d* = −0.38, 95%CI (−1.32 to 0.60)]. In contrast, time-trial performance (*p* = 0.35) and v4_[bla]_ (*p* = 0.89) were independent of the period of training. Table 5 documents the percentage change in each parameter of performance from before to after each period of training.

**Table 5:** Back-transformed means (in %) ± standard deviations for the percentage of each parameter related to performance and training period.

	**Training period**		
	**PP1**	**PP2**	**CP**
**Parameter**			
v2_[bla]_	3.62 ± 3.43^*^	–0.84 ± 2.16	2.67 ± 2.37
	(*n* = 10)	(*n* = 7)	(*n* = 8)
v4_[bla]_	2.63 ± 2.82	2.72 ± 2.24	1.40 ± 3.86
	(*n* = 10)	(*n* = 7)	(*n* = 8)
VO_2peak_	–3.11 ± 5.94^*^	25.48 ± 11.38^†^	0.34 ± 12.06
	(*n* = 9)	(*n* = 6)	(*n* = 8)
1,500-m time-trial	–0.16 ± 4.12	–3.46 ± 3.53	–0.38 ± 4.45
performance	(*n* = 10)	(*n* = 7)	(*n* = 8)

* *Significantly different from PP2; significantly different from CP*.

The relationships between training variables and alterations in physiological parameters, as well as changes in performance in the 1,500-m time-trial are summarized in Table 6.

**Table 6:** Correlations between the differences in physiological characteristics and parameters related to performance before and after each of the three training periods (PP1, PP2, CP), on the one hand, and the time spent in each training zone, total time spent in all three zones, and the Polarization Index, on the other.

	**VO_2peak_**			**v2_[bla]_**			**v4_[bla]_**			**1,500-m time-trial performance**		
	***r***		***p***	***r***		***p***	***r***		***p***	***r***		***p***
	**95%CI [LL–UL]**			**95%CI [LL–UL]**			**95%CI [LL–UL]**			**95%CI [LL–UL]**		
Time in Zone 1 each week	−0.25		0.43	0.06		0.86	−0.41		0.16	**0.66**		**0.01**
	−0.76	–	0.45	−0.56	–	0.63	−0.81	–	0.25	0.10	–	0.90
Time in Zone 2 each week	0.37		0.24	−0.07		0.83	0.53		0.06	**−0.57**		**0.04**
	−0.34	–	0.81	−0.64	–	0.55	−0.10	–	0.86	−0.87	–	0.05
Time in Zone 3 each week	0.40		0.20	−0.17		0.59	0.18		0.56	−0.36		0.22
	−0.31	–	0.82	−0.70	–	0.48	−0.47	–	0.70	−0.79	–	0.30
Total time in Zones 1–3 each week	−0.25		0.43	0.06		0.86	−0.41		0.16	**0.66**		**0.01**
	−0.76	–	0.45	−0.56	–	0.63	−0.81	–	0.25	0.10	–	0.90
Polarization Index	0.12		0.71	0.13		0.68	−0.21		0.49	0.22		0.46
	−0.55	–	0.70	−0.51	–	0.68	−0.72	–	0.44	−0.43	–	0.73

*1,500-m time-trial performance, performance in connection with the all-out 1,500-m trial during the incremental step test; VO_2peak_, peak oxygen uptake; v2_[Bla]_, velocity associated with a blood lactate concentration of 2 mmol·L^−1^; v4_[Bla]_, velocity associated with a blood lactate concentration of 4 mmol·L^−1^; 95%CI [LL–UL], p, significance values; r, correlation values; 95% Confidence Interval Lower Limit – Upper Limit. Significant correlations are highlighted in bold*.

## Discussion

The current retrospective investigation was designed (i) to describe the TID of elite sprint kayakers and a canoeist during the two different preparatory, as well as the competitive period of a single training season and (ii) to evaluate the relationship between the distribution of time spent in each training zone and alterations in parameters related to performance. To the best of our knowledge, this is the most thorough study on these topics to be reported to date.

### Seasonal Analysis of the Distribution of Training Intensity

#### Observation at the Group Level

The present observation that the training intensity of our group of athletes was pyramidal in structure throughout the entire season is in accordance with other reports on the large amounts of time spent in Z1 by rowers (Steinacker et al., 1998; Guellich et al., 2009; Nybo et al., 2014; Plews et al., 2014; Treff et al., 2017), cyclists (Lucía et al., 2000; Schumacher and Mueller, 2002; Zapico et al., 2007; Neal et al., 2011), triathlon athletes (Neal et al., 2013), cross-country skiers (Torvik et al., 2021) and runners (Esteve-Lanao et al., 2005). One explanation for this finding is that low-intensity training is needed to counteract potential negative effects (e.g., autonomic and hormonal stress, energy depletion) of training at intensities at or above threshold intensity (Bourgois et al., 2019).

In contrast, two prospective examinations of the TID of elite Spanish sprint kayakers describe an emphasis on Z2 and Z3, with a block of high-intensity peaking designed to improve submaximal and maximal performance (García-Pallarés et al., 2009, 2010). The first of these studies involved 12 weeks of such block periodization and the follow-up study this same 12-week block periodization in combination with 22 weeks of linear periodization divided into three periods of training. The first period of each model was designed to improve the anaerobic threshold, the next period aimed to improve maximal aerobic power and the final period was meant to improve specific race pace. These two investigations found that both models of periodization improve all parameters related to performance, with similar enhancement in VO_2peak_ (9.0–9.9%) and VO_2_ at the anaerobic threshold (7.8–8.6%) and more pronounced improvement in paddling speed at VO_2peak_ with block periodization (5.8 vs. LP: 3.3%).

The markedly higher proportions of their training that sprint kayakers spend in Z2 and Z3 in comparison to athletes involved in other sports might reflect differences in the type of muscular work being performed. During kayaking, propulsion is generated primarily by the upper-body musculature, whereas sports such as running, cycling, rowing, skiing, etc., involve primarily lower-body or even whole-body work for propulsion. For example, while using a cycle ergometer, kayakers can sustain an intensity of arm cranking at VO_2max_ significantly longer than cyclists (Billat et al., 1996).

These differences also lead to differences in the types of biomechanical stress (i.e., force generation by muscles and joints, frequency of movement, impact on muscles and joints, etc.) associated with sports that involve primarily the legs or arms and/or with sports that entail weight-bearing and those that do not, which may help explain differences in TID (Bourgois et al., 2019; Sandbakk et al., 2021). In addition, differences in cardiorespiratory and vascular demands, as well as metabolic load and/or the types of muscle fibers recruited may also contribute to dissimilarities in TID (Bourgois et al., 2019). Moreover, with Olympic races lasting from no more than 35 s (200 m) to ~240 s (1,000 m), kayaking and canoeing sprints place greater short-term demands on the athlete than do sports such as road cycling, long-distance running, and cross-country skiing. Nonetheless, our observation that over the course of the season the pyramidal TID of elite kayakers and canoeists tends to become more polarized, with a substantial increase in the time spent in Z1 and less in Z2 and Z3, is in accordance with earlier reports on elite athletes participating in a variety of endurance sports, including rowing (Guellich et al., 2009; Plews et al., 2014), cross-country skiing (Sandbakk et al., 2011; Tønnessen et al., 2014; Solli et al., 2017), running (Robinson et al., 1991) and cycling (Lucía et al., 2000) (Figure 3D).

This successive change in TID was accompanied by a 1.5-h increase in the total training time per week. In their study on young world-class rowers, Guellich et al. (2009) observed a similar pattern and suggested that, not unexpectedly, this increase requires more sessions of low-intensity exercise in order to avoid excess fatigue and overtraining. However, in our case, this shift to more low-intensity training only occurred during the CP, even though the total volume of training in PP2 was already higher than in PP1.

It is interesting to speculate that this increase in the overall volume of specific training without corresponding reductions in the amounts of Z2 and Z3 might reflect the greater opportunity for physical and psychological recovery associated with the COVID-19-lockdown during PP2. In this context, comparison of 4-week periods before and after this lockdown was imposed indicated that during the lockdown more sleep and time spent lying down, in combination with less moderate-to-vigorous physical activity improved recovery for at least certain athletes (Zinner et al., 2020). Improvements in sleep are known to exert a particularly positive effect on both recovery and performance (Watson, 2017). This proposal is in line with recent research stressing the importance of a more holistic approach to the factors that influence performance (e.g., life stress, sleep, daily physical activity, nutrition, etc.) when analyzing the effects of training, including the distribution of training intensity, on athletes (Sperlich and Holmberg, 2017; Kiely, 2018).

#### Observations on Individual Athletes

**Figure 5:**
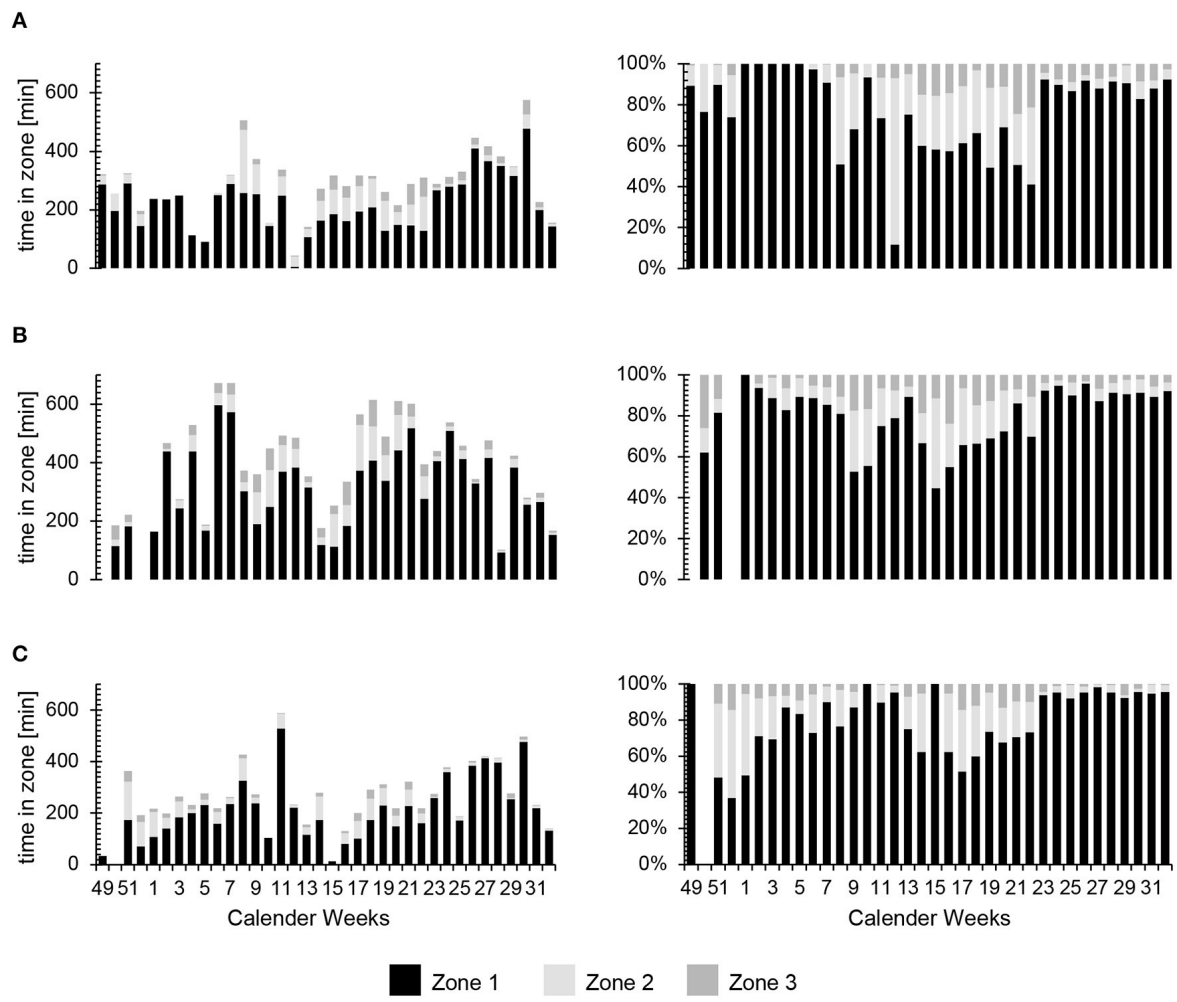
Representative values for the absolute (min) and relative (%) amounts of time spent in the different training zones during each week of the period of observation. **(A)** Participant 7, **(B)** Participant 4 and **(C)** Participant 11.

In contrast to the overall changes in TID as the season progressed, assessment of individual athletes resulted in a more heterogeneous pattern (Figures 3B–D). For example, during PP1 subjects 1, 7, 10, and 12 performed extremely large amounts of training in Z1 (as much as 94%) and extraordinarily little to almost none in Z2 and Z3; while others spent markedly less time in Z1 (e.g., 61 and 68% in the case of subjects 8 and 11, respectively) and more time in Z2 and Z3. Furthermore, even for one and the same athlete, the volume of training and distribution of its intensity varied considerably week-to-week throughout the entire season (cf. Figures 5A–C).

This extensive inter- and intra-athlete variability raises questions concerning the interpretation of group means and patterns. Such pronounced individual differences in TID patterns (Bourgois et al., 2019) may reflect, among other factors, differences in genetic make-up, biomechanical characteristics, physiological profile, biological age, training age and experience, off-training physical activity, recovery strategies, environmental conditions and psychological stress (Sperlich and Holmberg, 2017; Kiely, 2018). Overall, successful coping with stressors by the individual athlete probably plays a more important role in optimizing both short- and long-term performance than any particular distribution of training intensity during any given period of training.

### Seasonal Changes in Parameters Related to Performance

Although the only statistically significant alterations in parameters related to performance observed here were a more pronounced increase in v2_[BLa]_ (+3.6%) following PP1 than after PP2, but not after CP, and a greater elevation in VO_2peak_ (± 25.5%) after PP2 than following either PP1 or CP (Table 5), our results are to a certain extent comparable to previous reports. For instance, the improvement in v2_[BLa]_ and v4_[BLa]_ during PP1 are comparable to those observed by García-Pallarés et al. (2009, 2010). However, after a period without training, the sprint kayakers in these other investigations could elevate their paddling speed at the second ventilatory threshold by 2.2% through training in blocks during which 55–67% of the overall time was spent at or above an intensity corresponding to the anaerobic threshold (Z3). In contrast, our athletes, who only trained 4% of the time at intensities above the anaerobic threshold (Z3) and 12% at threshold intensities (Z2), exhibited a similar increase (~2.6%).

Previously, Ingham et al. (2008) found that in the case of rowers, more training at or above threshold intensity improved performance less than a large amount of low-intensity training at the lactate threshold. This observation is in accordance with indications that training more than 20% of the time at Z2 may have a negative impact on the autonomic nervous system (Esteve-Lanao et al., 2007), leading to less favorable adaptation of performance, potentially due to stress and the likelihood of down-regulation of the sympathetic nervous system in response to a large volume of high-intensity exercise (Esteve-Lanao et al., 2007). As mentioned above, extensive training might produce a need for a high proportion of low-intensity exercise designed to avoid excessive fatigue (Guellich et al., 2009). However, the sprint kayakers involved in the studies by García-Pallarés et al. (2009, 2010) had trained longer and performed better than our subjects. Thus, even small improvements in the performance of those athletes may have been more difficult to achieve, requiring more high-intensity training.

Our findings indicate that PP2 elicited improvements in v4_[BLa]_ (2.7%), VO_2peak_ (25.5%) and 1,500-m time-trial performance (3.5%), probably by increasing the training time spent in Z3 at the expense of Z1. Previous research has shown that higher proportions of Z3 may elicit superior adaptations that improve performance (Driller et al., 2009; Chéilleachair et al., 2017). In our case, the extraordinary restrictions imposed by the COVID-19 pandemic during this training period could possibly have augmented such exceptional adaptations, conceivably by allowing more recovery from training. In comparison, with higher proportions of training intensities at or near VO_2peak_ during two blocks 5 and 6 weeks long, elite Spanish sprint kayakers improved their VO_2peak_ by 5–7%, with no change in velocity at the anaerobic threshold or paddling speed at VO_2peak_ during an incremental test on a kayak ergometer (García-Pallarés et al., 2009, 2010). It is worth noting that the differences in air and water temperature between testing at T2 and T3 may have had an influence on our results as well.

During the CP we observed additional improvement in the velocity at both the anerobic (1.4 %) and aerobic thresholds (2.7 %). The lack of marked changes in VO_2peak_ (0.3%) or 1,500-m time-trial performance (0.4%) is comparable to the findings by Treff et al. (2017) in connection with an 11-week period of preparation for competition by elite German rowers. With a TID comparable to that of our athletes, these rowers exhibited no change in VO_2peak_ or 2,000 m-TT performance, but, in contrast to our present observations, no alteration in power with a blood lactate concentration of 2 or 4 mmol·L^−1^ either. In contrast, following tapering phases 2 (García-Pallarés et al., 2009) or 4 weeks in duration (García-Pallarés et al., 2010), the paddling speed of elite sprint kayakers at VO_2peak_ during an incremental test on a kayak ergometer was improved, with no changes in any physiological parameters related to performance. This difference might reflect the fact that 45–47% of the training by those kayakers was in Z3, compared to only 4% in our case, and/or to the different incremental test employed by the Spanish researchers.

It should be noted that our relatively small study population probably reduced the ability to confirm the statistical significance of small changes in the parameters monitored. A recent analysis concluded that changes as small as ~0.3–0.6% in the performance of international canoeists and kayakers improves their chances of winning a medal substantially (Bonetti and Hopkins, 2010). Thus, even the 0.4% improvement in 1,500-m time-trial performance following the CP observed here may be of significance.

### Correlations Between the Distribution of Exercise Intensity and Parameters Related to Performance

In addition, the time spent in Z1 and total training time in all three zones demonstrated a pronounced positive correlation with the improvement in 1,500-m time-trial performance. This indicates that if, at the same time, the overall volume of training is reduced, the improvement is derived from Z2 training only, which agrees well with an earlier report (Esteve-Lanao et al., 2007). This finding may reflect the negative impact of Z2 training on the autonomic nervous system (e.g., increased stress) discussed above. Of relevance to these considerations is our observation that changes in TID from PP2 to CP decreased the proportion of time spent in Z2 from 12±7% to 5±4%, indicating that adaptations in response to Z2 exercise intensity are not beneficial during the phase of competition, at least not in connection with canoe sprinting.

When interpreting the correlations observed, it is important to be aware of the limitation that we included neither the TID of non-specific training (e.g., strength training, dry-land endurance training, etc.) nor off-training variables in our analysis.

## Limitations

Certain limitations associated with the current investigation warrant consideration. The relatively small number of subjects, as well as the fact that data for certain individual periods of training were missing almost certainly affected our statistical analysis, especially with respect to a potential type-II error. At the same time, it is difficult to conduct investigations of this nature on large numbers of highly trained athletes, who must prioritize their own individual training and competition. Furthermore, although we chose to include one canoeist since the training of sprint canoeists and kayakers in Germany is similar, the differences in the biomechanical characteristics of and muscle groups involved in these two sports may have affected the results obtained. Moreover, it is important to note that with about half of the training being performed on land (Figure 2), analysis of TID on-water does not cover all of the stress to which these athletes are exposed. More general training (e.g., resistance training, endurance training on land, etc.), as well as various additional factors (e.g., sleep, daily physical activity, nutrition, etc.) must also be taken into account in connection with holistic evaluation of an athlete's training stress.

It should be noted that monitoring training on the basis of HR, with its delayed response at the beginning of a session of exercise, may underestimate the extent of high-intensity training and overestimate the time spent training in the threshold zone (Hogan et al., 2019, 2020). This is especially true in the case of very short high-intensity sessions (<30 s), in which HR_max_ may never be reached (Seiler, 2010; Buchheit and Laursen, 2013). Thus, it is highly likely that the time spent in Z3 during the CP observed here is an underestimation.

Furthermore, Hogan et al. (2019) recently highlighted the fact that during sessions of endurance training, analysis on the basis of HR indicates that less time is spent at < LT_2_ and more time at ≥LT_2_ than indicated by measurement of power. This difference reflects the cardiovascular drift associated with prolonged exercise of mild-to-moderate intensity, a drift observed earlier in connection with other endurance activities as well (Achten and Jeukendrup, 2003; Vogt et al., 2006; Nimmerichter et al., 2011). Thus, during PP1 it is most likely that the proportion of Z1 was underestimated, while the proportion of Z3 overestimated here. In the future, these problems associated with the use of HR as a measure of intensity should be recognized and additional external measures of training intensity, such as speed or power, included. Unfortunately, this was not possible to do in the present study.

According to the coaches of our athletes, their overall approach to training was not altered significantly by the restrictions introduced to combat the COVID-19 pandemic. However, these coaches did report some restrictions of access to gyms, which resulted in fewer high-load sessions of resistance training by some of the athletes. Furthermore, cancellation of all national competitions (with the exception of the German national championships) may have led to changes in the structure of training, especially during the period of competition.

## Conclusion

Our seasonal analysis revealed extensive interindividual variation. Over the entire period of observation TID was pyramidal and all parameters related to performance improved. During PP2, when the COVID-19 lockdown was in place, the proportion of time spent in Z3 doubled, while that spent in Z1 was lowered; the total time spent training on water increased; these changes may have accentuated the improvement in performance during this period. A further increase in total on-water training time during CP was made possible by reductions in the proportions of time spent in Z2 and Z3, so that more fractions of time was spent in Z1.

## Data Availability Statement

The original contributions presented in the study are included in the article/supplementary materials, further inquiries can be directed to the corresponding author/s.

## Ethics Statement

The studies involving human participants were reviewed and approved by Julius Maximilians University Würzburg. Written informed consent to participate in this study was provided by the participants and/or the participants' legal guardian/next of kin.

## Author Contributions

MM, BS, and CZ: conceptualization and investigation. MM, RL, BS, CZ, and H-CH: data analysis. MM, BS, CZ, and H-CH: writing, review, and editing of manuscript. All authors contributed to the article and approved the submitted version.

## Funding

This project was supported financially by the German Federal Institute of Sports Sciences.

## Conflict of Interest

The authors declare that the research was conducted in the absence of any commercial or financial relationships that could be construed as a potential conflict of interest.

## Publisher's Note

All claims expressed in this article are solely those of the authors and do not necessarily represent those of their affiliated organizations, or those of the publisher, the editors and the reviewers. Any product that may be evaluated in this article, or claim that may be made by its manufacturer, is not guaranteed or endorsed by the publisher.
